# Evaluation of a *Rhodomyrtus tomentosa* ethanolic extract for its therapeutic potential on *Staphylococcus aureus* infections using in vitro and in vivo models of mastitis

**DOI:** 10.1186/s13567-019-0664-9

**Published:** 2019-06-20

**Authors:** Auemphon Mordmuang, Eric Brouillette, Supayang Piyawan Voravuthikunchai, François Malouin

**Affiliations:** 1 Department of Microbiology and Excellence Research Laboratory on Natural Products, Faculty of Science and Natural Product Research Center of Excellence, Prince of Songkla University, Hat Yai, Songkhla 90112 Thailand; 2 Centre d’Étude et de Valorisation de la Diversité Microbienne (CEVDM), Département de biologie, Faculté des sciences, Université de Sherbrooke, Sherbrooke, QC J1K 2R1 Canada; 3 School of Medicine, Walailak University, Tha Sala, Nakhon Si Thammarat 80160 Thailand

## Abstract

An ethanolic extract from *Rhodomyrtus tomentosa* leaves (RTL) was studied as a natural alternative to control *Staphylococcus aureus*, which is an important pathogen responsible for bovine mastitis. The minimal inhibitory concentrations (MICs) of the RTL extract and of rhodomyrtone, a pure compound isolated from the plant, were determined by a microdilution method. Rhodomyrtone and the RTL extract exhibited antibacterial activity against *S. aureus*, including its persistent phenotype (SCV: small-colony variant) and a biofilm hyperproducer strain, with MICs of 0.25–0.5 and 8–16 µg/mL, respectively. Time-kill kinetics showed a strong bactericidal activity for both the RTL extract- and rhodomyrtone-treated bacteria at 2 × MIC as early as 4 h post-exposure. An additive effect of the extract at 0.5 × MIC was observed in a combination with oxytetracycline or pirlimycin against *S. aureus* by showing a 64- to 128-fold reduction in antibiotic MICs. Moreover, the RTL extract significantly decreased the number of intracellular SCVs inside bovine mammary epithelial cells. However, the extract or its combination with pirlimycin only slightly improved the activity of pirlimycin against the bacterial colonization of mouse mammary glands. In vitro MICs determined in the presence of casein indicated that the limited activity of the RTL extract in the murine model of mastitis could be linked to neutralization of active components by milk proteins. While the RTL extract showed interesting antibacterial properties in vitro, to be considered as an alternative to antibiotics in dairy farms, formulation studies are needed to cope with the observed reduction of activity in vivo.

## Introduction

Bovine mastitis caused by *Staphylococcus aureus* is an important problem in the dairy industry worldwide. The bacterium is associated with subclinical and clinical bovine mastitis, that can spread contagiously to other animals in the herd [1–3]. *S. aureus* produces many toxins including extracellular hydrolytic enzymes that can impair host defenses and damage mammalian cells and tissues but one of the crucial functional mechanisms of *S. aureus* virulence is its ability to invade, persist and replicate inside host cells [4, 5].

*Staphylococcus aureus* strain Newbould, a prototypic strain isolated from a case of clinical bovine mastitis and its laboratory-derived *hemB*-deleted mutant strain, a small colony variant (SCV), have been extensively studied for their phenotype and role in persistent infections [6, 7]. The ability of SCVs to persist in phagocytic cells or in nonprofessional phagocytes such as fibroblasts, endothelial cells, and epithelial cells has been established [8–10]. Internalization of bacteria into host cells can facilitate evasion from the host immune system and is a phenomenon that is associated with the chronic or recurrent manifestation of the disease. Switching from the normal to the SCV phenotype and vice versa is believed to be part of the natural infection process for *S. aureus* [11]. In addition, clinical SCVs have been frequently isolated following antibiotic pressure [12, 13]. SCVs have been detected in persistent bovine mastitis [14] and a therapeutic solution for *S. aureus* bovine mastitis needs to be successful against the SCV phenotype.

Antibacterial therapies are being challenged by the widespread development of bacterial resistance. Bacterial strains designated as methicillin-resistant *S. aureus* (MRSA) have been isolated from bovine milk [15]. In addition to the occurrence of antibiotic resistant strains, which may be spread from cow to cow by contaminated equipment or in the community by farm workers, treatment of intramammary infections using antibiotics increases consumer concerns about food-related health risks and problems associated with drug residues in milk. Therefore, attempts of using natural alternatives to antibiotics for treatment of mastitis have been made, in order to minimize the usage of traditional antibiotics or chemical agents in the animal food industry [16]. The reduction of antibiotic use and discovery of alternatives in agri-food sectors where antibiotics are heavily used should directly benefit human health as it may limit and prevent the development of antibiotic resistance.

*Rhodomyrtus tomentosa* is a medicinal plant belonging to the *Myrtaceae* family and an ethanolic leaf extract of the plant was previously shown to have an antibacterial activity against Gram-positive bacteria including *S. aureus*, coagulase-negative staphyloccocci and *Streptococcus* spp., that are widely found as causative agents of bovine mastitis [17]. In addition, rhodomyrtone, a pure compound isolated from this plant, has demonstrated a strong inhibitory effect against *S. aureus* similar to that obtained with vancomycin, an important anti-*S. aureus* drug in human medicine [18]. Recently, we have reported that a *R. tomentosa* ethanolic leaf (RTL) extract affected *S. aureus* cell surface properties by increasing its hydrophobicity and consequently disturbed bacterial adhesion and invasion into host cells in an ex vivo bovine udder epidermal tissue model [19].

The use of a plant extract that is easy to produce could facilitate its introduction as a novel natural alternative to antibiotics. In the present study, the aims were to determine the effect of the RTL extract on the ability of *S. aureus* to invade bovine mammary epithelial cells, to specifically include *S. aureus* SCVs among the studied bacterial targets, and to investigate any possible synergistic effects the RTL extract could have with traditional antibiotics, in order to improve their therapeutic efficacy or to reduce the amounts needed. A mastitis-relevant mouse *S. aureus* intramammary infection model was used to evaluate in vivo efficacy.

## Materials and methods

### Bacterial strains and growth conditions

*Staphylococcus aureus* strains used in this study included Newbould (ATCC 29740) and its laboratory-derived isogenic small colony variant (SCVs) counterpart (∆*hemB*) that was described before [6]. Also included was strain SHY97-3906 that was isolated from a case of clinical bovine mastitis [20]. Other isolates were selected from the Mastitis Pathogen Culture Collection (MPCC) belonging to the Canadian bovine mastitis and milk quality research network (CBMQRN, St-Hyacinthe, QC, Canada), as described in detail in Reyher et al. [2]. These isolates included one bovine methicillin-resistant *S. aureus* (MRSA strain 1158, spa type t451, barcode 1081-2464), one biofilm hyperproducer (strain 2117, spa type t13401, barcode 1070-5001) evidenced in a previous study [21], as well as strains 2236 (spa type t257, barcode 2090-2582), and 2290 (spa type t529, barcode 2110-3742). Also from the collection were two coagulase-negative strains such as *S. simulans* 3100-0949 and *S. chromogenes* 3140-3115 that were used for antibacterial activity testing. The bacterial strains were maintained on tryptic soy agar or broth (TSA or TSB) as well as on mannitol salt agar (MSA), all from Becton–Dickinson (USA), and grown at 35 °C for 18–24 h.

### Preparation of *R. tomentosa* leaf (RTL) extract and rhodomyrtone

The leaves of *Rhodomyrtus tomentosa* (Aiton) Hassk. were collected from the Singha Nakhon district, in the Songkhla province (Thailand) by Dr. Asadhawut Hiranrat (Department of Organic Chemistry, Faculty of Science, Prince of Songkla University). The voucher specimen (A. Hiranrat 001) was identified by J. Wei and has been deposited in the herbarium of Department of Biology, Faculty of Science, Prince of Songkla University, Thailand [22]. *R. tomentosa* leaves were dried in an oven at 60 °C for 48 h and ground in an electric blender. Dried leaf powder was extracted with 95% ethanol at room temperature and kept in the dark for 1 week. The extract was evaporated for 48 to 72 h by a rotary evaporator (BUCHI Rotavapor R-114, Büchai Labortechnik AG, Flawil, Switzerland) until it was completely dry (visual inspection). The extract was then dissolved in dimethyl sulfoxide (DMSO, Sigma-Aldrich Inc., Oakville, Canada). Pure rhodomyrtone (Sigma-Aldrich Inc.) was also dissolved in DMSO and kept at −20 °C until used.

### Determination of the minimum inhibitory concentration (MIC) and minimum bactericidal concentration (MBC)

The minimum inhibitory concentrations (MICs) of the RTL extract, rhodomyrtone and other antibiotics were determined by a broth microdilution method according to the recommendations from the Clinical Laboratory Standards Institute (CLSI) for antimicrobial susceptibility testing [23]. The antibacterial agents, prepared in cation-adjusted Mueller–Hinton broth (CAMHB, Becton–Dickinson, Mississauga, Canada) to a final concentration of 256 µg/mL (highest concentration having ≤ 0.5% DMSO), were separately added and further diluted by twofold serial dilutions in 96-well plates. An equal volume of a bacterial suspension (~1 × 10^6^ colony-forming units (CFU)/mL) was added to each well containing the antibacterial agents and incubated at 35 °C for 18–24 h. In all assays, sterility and bacterial growth controls were included. Vancomycin (Sigma-Aldrich Inc.) and pirlimycin hydrochloride (Pirsue^®^, Zoetis Canada Inc., Kirkland, Canada) were included as reference drugs and for quality controls. The MIC was defined as the lowest concentration of drug yielding no visible growth. The minimum bactericidal concentration (MBC) was determined subsequent to the MIC assay. A 10-µL aliquot from the visually clear wells containing the MIC value and higher concentrations were spread onto TSA plates. The agar plates were incubated at 35 °C for 18 h. The MBC was defined as the concentration killing 99.9% of the initial inoculum. In some specified experiments, 5% casein (Sigma-Aldrich Inc.) was added to CAMHB to mimic the milk environment.

### Checkerboard assay and determination of the fractional inhibitory concentration (FIC) index

A two-dimensional broth microdilution checkerboard assay was performed to determine the MICs of the RTL extract or rhodomyrtone in combination with 10 antibiotics, including ampicillin, amoxicillin, penicillin, oxacillin, ceftiofur, ciprofloxacin, gentamicin, kanamycin, pirlimycin, and oxytetracycline against *S. aureus* Newbould. The antibiotics were serially diluted in the y-axis and the extract or rhodomyrtone in the x-axis. An equal volume of a bacterial suspension (~1 × 10^6^ CFU/mL) was added to each well containing the antibacterial agents and incubated at 35 °C for 18–24 h. In all assays, sterility and bacterial growth controls were included. The MIC was defined as the lowest concentration of drug (alone or in combination) yielding no visible growth. The antibacterial effect of each combination was determined by means of the FIC according to the following equation [24]:

$$\begin{aligned} {\text{FIC}}_{{\text{A}}} &= MIC_{{combination}} /MIC_{A} , \\ {\text{FIC}}_{{\text{B}}} &= MIC_{{combination}} /MIC_{B} , \\ \sum {\text{FIC }}& = {\text{ FIC}}_{{\text{A}}} + {\text{ FIC}}_{{\text{B}}} \\ \end{aligned}$$

The FIC index (∑FIC) was interpreted as follows: ∑FIC ≤ 0.5, synergism; > 0.5–1, additivity; > 1–4, indifference; ≥ 4, antagonism.

### Kill kinetics

The bactericidal activity of the RTL extract and rhodomyrtone against *S. aureus* was assessed by time-kill experiments. *S. aureus* Newbould and its isogenic Δ*hemB* mutant were used as representative strains for the wild-type and SCV phenotype, respectively. The bacterial inoculum (~1 × 10^6^ CFU/mL), as evaluated by turbidity using McFarland standards and confirmed by plating on agar for CFU counts, was grown with CAMHB supplemented with the plant ethanolic extract or rhodomyrtone at concentrations of 0.5×, 1×, 2×, and 4× their respective MIC value. The bacterial cultures were incubated at 35 °C with shaking and their growth was monitored over 24 h. Samples were collected at various time intervals followed by tenfold serial dilutions. A 10 µL aliquot was spread onto TSA plates for determination of the CFU. The bacterial growth kinetics was analyzed by plotting log 10 CFU/mL versus time.

### Cell culture

Bovine mammary gland epithelial cells (MAC-T) were cultured in growth medium composed of Dulbecco’s Modified Eagle’s Medium (DMEM, Wisent Inc., Saint-Jean-Baptiste, Canada) supplemented with 10% inactivated fetal bovine serum (FBS, Wisent Inc.), 1% sodium pyruvate, 10 µg/mL insulin, 5 µg/mL hydrocortisone, 100 units/mL penicillin, 100 µg/mL streptomycin, and 0.025 µg/mL of fungizone. The cells were passaged twice a week up to ten passages after being thawed from the stock culture and incubated in 5% CO_2_ at 37 °C. A confluent monolayer of the cell culture was treated with a 0.25% trypsin solution (Wisent Inc.) for 3–5 min and neutralized by adding DMEM supplemented with 10% FBS. Single cells were collected from the suspension after centrifugation using an Allegra™ 6R centrifuge (Beckman Coulter Canada LP, Mississauga, Canada) at 250 *g*, 25 °C, for 10 min. For specified experiments, aliquots of 250 µL of MAC-T cells were seeded into 24-well culture plates and incubated in the growth medium at 37 °C in a humidified incubator with 5% CO_2_, until the desired confluence was reached.

### Cytotoxicity test

The cytotoxicity of the RTL extract, rhodomyrtone or pirlimycin for MAC-T cells was quantified using a lactate dehydrogenase (LDH) assay according to the manufacturer’s instructions (LDH Cytotoxicity detection kit, Roche Diagnostics GmbH, Mannheim, Germany). The day before the experiment, the suspension of MAC-T cells was adjusted to 2 × 10^5^ cells/mL in the growth medium and 250 µL of the cell suspension were added into 48-well tissue culture plates. The plates were incubated at 37 °C for 24 h in a humidified incubator with 5% CO_2_ to obtain a confluent monolayer (~1 × 10^5^ cells/well). The cell monolayer was washed twice with DMEM and the growth medium was replaced with the assay medium (DMEM supplemented with 1% FBS without antibiotics) containing different concentrations of the tested agents. A 1% triton X-100 solution (Sigma-Aldrich Inc.) and the assay medium itself were used as a high cytotoxicity control and the low cytotoxicity control, respectively. Cell-free supernatants were separately collected after treating for 8–12 h. An aliquot of 100 µL from each tested and control well were transferred to a 96-well plate, followed by adding 100 µL of the LDH reaction mixture. The LDH reaction in each sample was measured by the optical density (OD) at 490 nm/660 nm using a spectrophotometric plate reader. The experiments were performed in triplicate. Percentage of cytotoxicity of antibacterial compounds were determined and calculated according to the formula provided by the manufacturer, as follows:

$${\text{Cytotoxicity}}\left( \% \right) = \left[ {\left( {{\text{OD}}\;{\text{of}}\;{\text{the}}\;{\text{tested}}\;{\text{cell}}\;{\text{mixture }} - {\text{ OD of the compound alone}}} \right) - {\text{ OD}}\;{\text{low}}\;{\text{control}}} \right] / \, \left[ {\left( {{\text{OD}}\;{\text{high}}\;{\text{control }} - {\text{ OD}}\;{\text{low}}\;{\text{control}}} \right)} \right] \times 100$$

### Bacterial invasion assay

MAC-T cells were adjusted to 5 × 10^4^ cells/mL with growth medium and 500 µL of the cell suspension was added into 24-well tissue culture plates. The plates were incubated at 37 °C for 48 h to obtain a >80% confluent monolayer (~1.5 × 10^5^ cells/well). The cell monolayer was washed twice with DMEM, shielded with 500 µL of invasion medium (DMEM supplemented with 1% FBS without antibiotics), and re-incubated for 4 h. The cell monolayers were then washed with DMEM and the cell culture medium was replaced with 500 µL of the freshly prepared bacterial suspension at a multiplicity of infection (MOI) of 10 or 100. Bacterial cell suspensions were prepared by adding an overnight culture of *S. aureus* into fresh TSB (1:20) containing ½ × MIC of the RTL extract or rhodomyrtone and incubated at 35 °C for 2 h. Vancomycin (also at ½ × MIC) was used as the reference drug. All cultures contained 0.25% DMSO. These pretreated bacterial cells were then washed twice with cold sterile PBS and the bacterial suspension was adjusted to 3 × 10^6^ CFU/mL in the invasion medium. After bacteria were added, each well was filled to 1 mL by adding 500 µL of the test antibiotics at half its MIC. The plates were incubated at 37 °C for 3 h. Unattached and extracellular bacteria were then removed from the monolayers by washing twice with DMEM and possible residual extracellular bacteria were eliminated by adding 700 μL of 2 µg/mL lysostaphin (Sigma-Aldrich). The culture was further incubated for 30 min, therefore, the total incubation period was subsequently 3.5 h. After incubation, the cell monolayers were washed twice with D-PBS (Wisent Inc, Canada), trypsinized with 100 µL of trypsin solution for 10 min, and lysed with 900 µL of sterile distilled water for 5 min. The cell lysates were mixed thoroughly to release intracellular bacteria. The number of CFU was determined by the standard colony counting technique on TSA. Data are presented as the percentage of bacteria that were internalized relative to the initial bacterial suspension exposed to MAC-T cells.

### Efficacy in a mouse mastitis model

An in vivo study was carried out using a mouse mastitis model according to the method described previously [25] with few modifications. Briefly, CD-1 lactating mice (Charles River, St-Constant, Canada) were used 12 to 14 days after off spring birth. The pups were removed 1 h before challenged with 100 μL of *S. aureus* strain 2117 or Newbould (200 CFU/gland) via intramammary inoculation in both the L4 (on the left) and R4 (on the right) abdominal mammary glands. Mammary glands were treated (intramammary administration) with the RTL extract (300 µg per dose, 2 doses to achieve maximal concentration), with pirlimicin (1 µg per dose) or a combination of both. For the extract, treatment was done at 0 and 4 h post-infection, whereas pirlimycin was administered at 4 h. PBS was used for the untreated control. The mammary glands were collected and homogenized at 16 h post-infection. The samples were serially diluted with PBS and plated on TSA and MSA for determination of the number of surviving bacteria. The data represent two independent experiments using several mice and glands in each. The animal experiments were conducted following the guidelines of the Canadian Council on Animal Care and approved by the institutional ethics committee on animal experimentation of the Faculté des sciences of Université de Sherbrooke.

### Statistical analysis

In this study, the data were analyzed using the GraphPad Prism Software (v.6.00). Tests used for the analysis of each experiment are specified in the figure legends.

## Results

### MIC and MBC of the RTL extract and rhodomyrtone

The RTL extract showed an antibacterial activity against a series of *S. aureus* and coagulase negative staphylococci (CNS) strains associated with bovine mastitis and had MIC values ranging from 8 to 16 μg/mL (Table 1). Noteworthy, the target strains included a *S. aureus* SCV (Newbould Δ*hemB*), a bovine MRSA and also a *S. aureus* of the spa type 13401 (strain 2117), producing a significant amount of biofilm [21]. The inhibitory spectrum was similar to that of rhodomyrtone, which however exhibited a stronger antibacterial activity with MIC values that ranged from 0.25 to 0.5 μg/mL. The pure compound provided inhibitory activity that was very close to the activity of vancomycin and pirlimycin, which had MIC values ranging from 0.25 to 1 μg/mL (Table 1). The MBCs of the RTL extract ranged from 16 to 128 μg/mL (i.e., 2–16 times the MIC), whereas rhodomyrtone showed MBCs that were 1–4 times its MIC.

**Table 1:** **Minimal inhibitory concentrations (MICs) and minimal bactericidal concentrations (MBCs) of the** ***Rhodomyrtus tomentosa*** **leaves (RTL) extract, rhodomyrtone, and reference antibiotics against staphylococcal strains**

Bacterial strains	MIC/MBC (µg/mL)			
	RTL extract	Rhodomyrtone	Pirlimycin	Vancomycin
Newbould (ATCC 29740)	16/128	0.5/0.5	0.5/1	0.5/1
Newbould ∆*hemB* (SCV)	8/16	0.5/1	0.25/0.5	0.5/1
*S. aureus* 1158c (bovine MRSA)	16/64	0.5/1	0.5/1	0.5/1
*S. aureus* SHY97-3906	8/16	0.5/1	0.5/1	0.5/1
*S. aureus* 2117	16/128	0.5/1	1/2	0.5/1
*S. aureus* 2236	16/64	0.5/1	1/2	0.5/1
*S. aureus* 2290	16/32	0.5/1	0.5/1	0.5/0.5
*S. simulans* 3100-0949	16/128	0.5/1	0.5/1	0.5/1
*S. chromogenes* 3140-3115	16/32	0.25/1	0.25/0.5	0.25/0.5

### Kill kinetics

**Figure 1:**
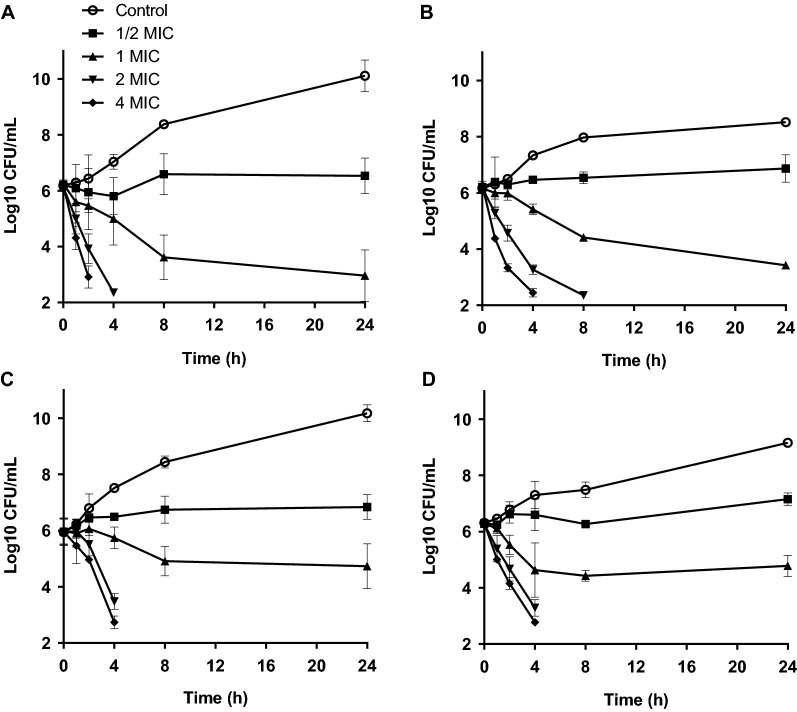
**Time-kill kinetics of the** ***Rhodomyrtus tomentosa*** **leaves (RTL) extract (A, B) and rhodomyrtone (C, D) against** ***S. aureus*** **Newbould (A, C) and its small-colony variant (SCV, Δ*hemB*) (B, D).** The data are expressed as means of three independent experiments. The detection limit was 100 CFU/mL.

The kinetic of bacterial killing by the plant extract and rhodomyrtone was determined by following bacterial counts over 24 h. The agents were used at a sub-inhibitory concentration, at the MIC, and at supra-MICs against *S. aureus* Newbould (Figures 1A and C) and against its SCV Newbould Δ*hemB* (Figures 1B and D). The RTL extract at ½ × MIC and 1 × MIC inhibited growth of the *S. aureus* by prolonging the bacterial lag phase. A strong bactericidal effect that decreased the number of viable bacterial cells by more than 3 log10 CFU/mL was observed using concentrations above the MIC against both strains within 8 h (Figures 1A and B). Treatment with rhodomyrtone showed almost identical kill kinetics than that of the RTL extract at the same multiples of its own MIC, although the concentrations used for the pure compound were much lower (Figures 1C and D).

### Cytotoxicity

The RTL extract showed a relatively low cytotoxicity on MAC-T cells, which slowly increased in a concentration dependent manner (Table 2). This was similar to that observed with pirlimycin, which is abundantly used for the treatment of bovine mastitis. For the RTL extract, there was ~10–12% cytotoxicity at the MIC against *S. aureus* strains (i.e., 8–16 μg/mL). Interestingly, rhodomyrtone was the least cytotoxic at concentrations of 1–32 µg/mL but its cytotoxicity sharply increased to 42 and 82% at concentrations of 64 and 128 µg/mL, respectively (Table 2). Noteworthy, those concentrations of rhodomyrtone represent 128 and 256 times, respectively, the MIC of this pure compound against *S. aureus* (Table 1). Note that, all antibacterial agents tested were diluted and tested in the presence of 3.2% DMSO (final concentration) and the solution of 3.2% DMSO that was used as an untreated control showed no toxicity on the MAC-T cell (i.e., no different than the no DMSO control; data not shown).

**Table 2:** **Cytotoxicity of the** ***Rhodomyrtus tomentosa*** **leaves (RTL) extract on MAC-T cells as determined by a LDH assay**

Concentration (µg/mL)	% cytotoxicity of antibacterial agents^a^		
	*R. tomentosa* extract	Rhodomyrtone	Pirlimycin
1	6.2 ± 1.4	0.2 ± 0.7	11.5 ± 1.4
2	6.9 ± 0.5	0.2 ± 0.2	14.4 ± 3.2
4	10.5 ± 0.6	0.2 ± 0.2	16.2 ± 1.3
8	10.9 ± 1.1	0.3 ± 0.5	18.9 ± 0.9
16	12.1 ± 0.04	0.4 ± 0.1	20.8 ± 0.5
32	12.7 ± 0.1	0.4 ± 0.3	23.5 ± 1.9
64	14.5 ± 1.8	42.2 ± 0.6	26.6 ± 3.0
128	16.4 ± 3.2	81.9 ± 3.2	28.3 ± 2.7

^a^The % cytotoxicity was calculated as described in “Materials and methods” section. Values are means of three independent experiments with standard deviations. All compounds were diluted and tested in presence of 3.2% DMSO (final concentration).

### Inhibition of *S. aureus* internalization into MAC-T cells

**Figure 2:**
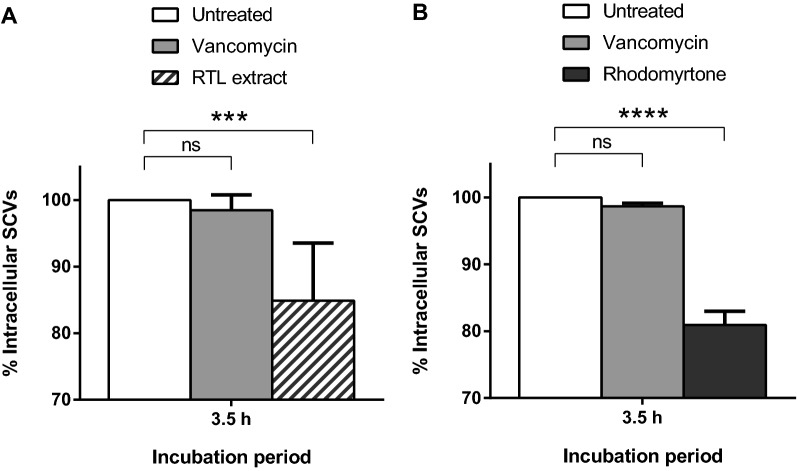
**Effect of the** ***Rhodomyrtus tomentosa*** **leaves (RTL) extract (A) and rhodomyrtone (B) at half their minimal inhibitory concentrations (MICs) on the internalization of the small-colony variant (SCV) strain Newbould Δ*hemB*** **into MAC-T cells after a 3.5** **h treatment.** Vancomycin was also used as a control in each assay. The bars represent the means of three independent experiments with standard deviations. One-way analysis of variance was performed with Dunnett’s post-test: ***P = 0.0002; ****P = 0.0001; ns: not significant (*P* > 0.05).

The number of bacteria internalized into MAC-T cells was first determined. As expected and shown before [7], at a MOI of 10 or 100, internalization of the Newbould SCV strain was always significantly greater than that of the prototypical Newbould strain (data not shown). For example, at a MOI of 10, approximately 6 × 10^6^ CFU/mL of the SCV strain were recovered from MAC-T cells after 3.5 h and these conditions were used to evaluate the inhibitory activity of the RTL extract on the internalization process. As shown in Figure 2, the presence of the RTL extract or the pure rhodomyrtone compound (both used at half their MIC) in the extracellular milieu together with the SCV strain, reduced the number of internalized bacteria to approximately 85 and 81% after 3.5 h, respectively, compared to that found for the untreated control (Figures 2A and B). Vancomycin, also used at half its MIC, showed no significant reduction of the SCV numbers internalized into MAC-T cells compared to the untreated control cells (Figures 2A and B). Noteworthy, none of the antibacterial agents used here at half their MICs reduced the amounts of viable bacteria in the cell culture medium for an incubation period of 3.5 h in the absence of MAC-T cells or in the CAMHB medium as seen in time-kill kinetic assays (Figures 1B and D). The decrease in SCV internalization by the RTL extract or rhodomyrtone can therefore not be due to bacterial killing prior to internalization into MAC-T cells.

### Benefits of compound combinations

In a checkerboard assay, multiple dilutions of 10 different antibiotics were combined with increasing concentrations of the RTL extract or rhodomyrtone, and a FIC index was determined for each tested combination against *S. aureus* Newbould for detection of possible synergy between some antibiotics and the natural products. For most antibiotics, no synergy was recorded. The exceptions were pirlimycin and oxytetracycline, which when combined with half the MIC of the RTL extract, the MIC of the antibiotic dropped by 64 (oxytetracycline) to 128 (pirlimycin) times (Table 3). The resulting FIC index was ~0.5 (0.508–0.516), which technically depicts an additive effect at the limit of synergism (≤ 0.5). Rhodomyrtone, which is already as potent as most of the antibiotics tested did not show any synergy against that *S. aureus* strain (FIC index of 1.01).

**Table 3:** **Minimal inhibitory concentrations (MICs, µg/mL) and the fractional inhibitory concentration index (∑FIC) of the** ***Rhodomyrtus tomentosa*** **leaves extract (RTLex) in combination with antibiotics (ATB) against** ***S. aureus*** **strain Newbould**

Antibiotic	Individual MIC^a^ ATB/RTLex	MIC in combination^b^ ATB/RTLex	MIC fold improvement^c^ ATB/RTLex	∑FIC^d^
Pirlimycin	0.5/16	0.004/8	128/2	0.508
Oxytetracycline	0.5/16	0.008/8	64/2	0.516

^a^MIC of the antibiotic (ATB) and MIC of the RTL extract (RTLex) when used alone.

^b^MIC of the ATB and MIC of the RTLex when used in combination.

^c^The fold improvement of the MIC is the ratio of the MIC alone/MIC in combination for the ATB or the RTLex.

^d^A ∑FIC of ≤ 0.5 is defined as synergism; 0.5–1 as additive; > 1–4 as indifference; > 4 as antagonism.

**Figure 3:**
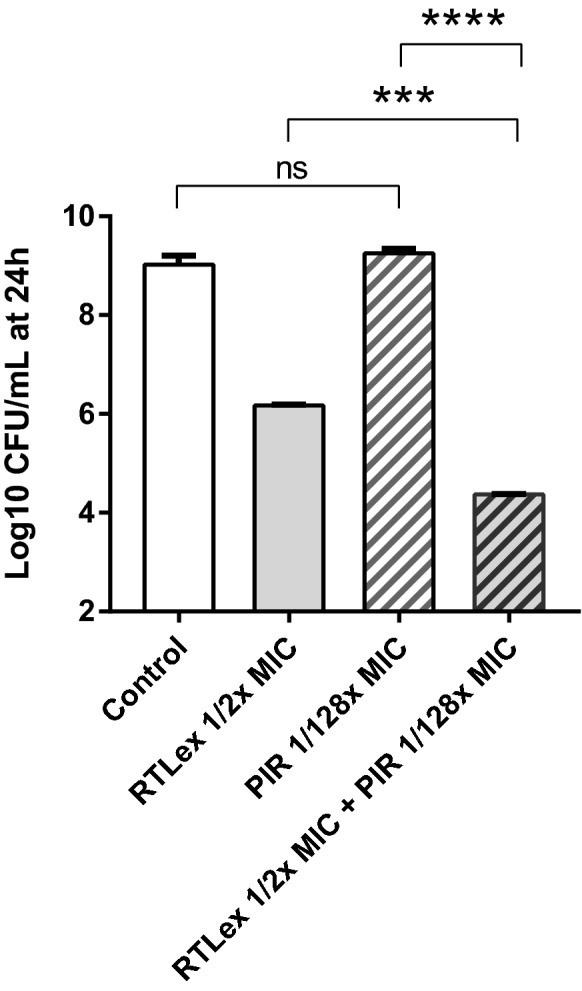
**Bactericidal activity of sub-inhibitory concentrations of the** ***Rhodomyrtus tomentosa*** **leaves extract (RTLex) and pirlimycin (PIR) used alone or in combination against** ***S. aureus*** **Newbould after 24** **h.** The bars represent the means of three independent experiments with standard deviations. Two-way ANOVA and Tukey’s multiple comparisons test: ****P < 0.0001; ***P = 0.0002; ns: not statistically significant (*P *> 0.05).

Since the RTL extract was bacteriostatic at half its MIC (Figures 1A and B), the bactericidal activity of the RTL extract in combination with a fraction of the pirlimycin MIC was evaluated to see if the additive effect or weak synergy observed in the checkerboard assay translated into killing. Figure 3 shows indeed that the combination of half the MIC of the RTL extract with 1/128 the MIC of pirlimycin kills significantly more than either of these agents used alone (*P* = 0.0002 and *P* < 0.0001, respectively). These results show that the RTL extract could be employed to drastically reduce the useful concentration of an antibiotic like pirlimycin.

### Efficacy of the RTL extract alone or in combination with pirlimycin in vivo

**Figure 4:**
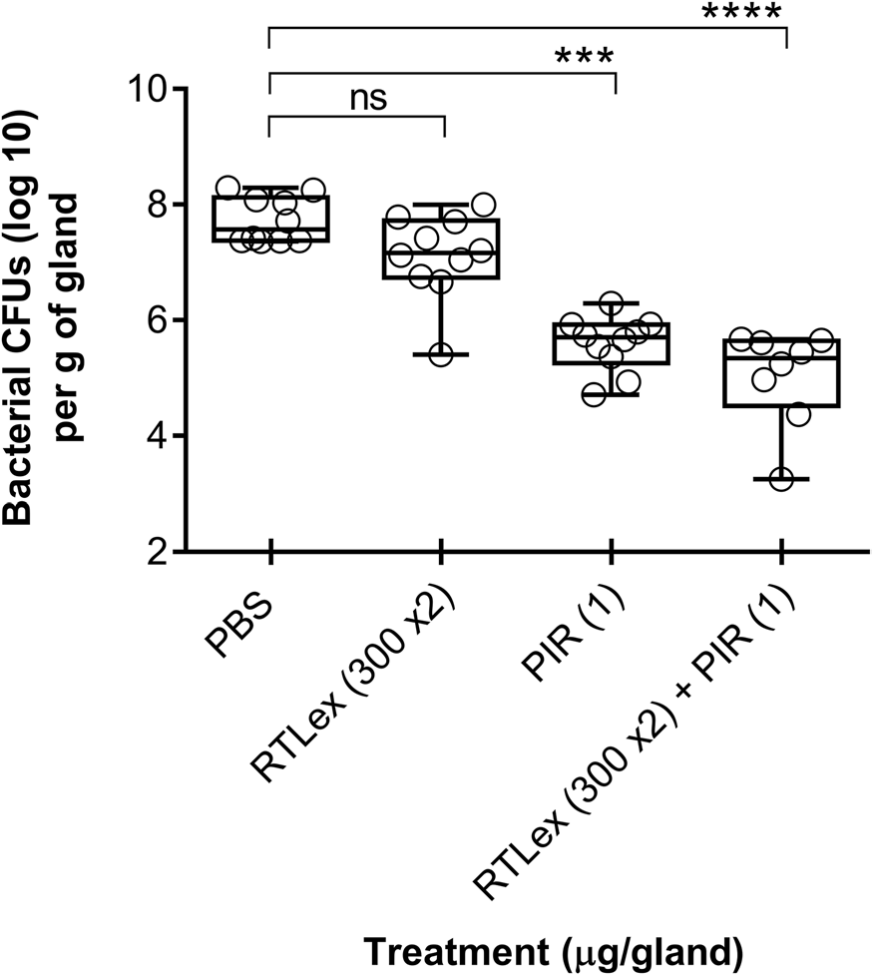
**Bacterial colony-forming unit (CFU) counts obtained from mice mammary glands 16** **h post-infection with** ***S. aureus*** **2117.** Mammary glands were treated (intra-mammary administration) with the *Rhodomyrtus tomentosa* leaves extract (RTLex: 300 µg per dose, 2 doses), with pirlimycin (PIR: 1 µg per dose), or a combination of both. For the extract, treatment was done at 0 and 4 h post-infection, whereas pirlimycin was administered at 4 h. PBS was used for the controls (animals infected but not treated). Each dot represents the CFU of individual glands (*n* = 8–10) and the median value for each group is indicated by the bar. Statistical differences between CFUs recovered from treated and untreated animals are shown (non-parametric Kruskal–Wallis ANOVA with Dunn’s post-test: ***P < 0.001; ****P < 0.0001; ns: not statistically significant (*P *> 0.05) vs. untreated control).

In vivo experiments were carried out to determine the therapeutic effects of the RTL extract, pirlimycin and a combination of both against a *S. aureus* challenge in the mouse mastitis model. The biofilm hyperproducing strain *S. aureus* 2117 was used for the challenge. The number of bacterial cells inside the mammary glands in the control condition (PBS treatment) reached a maximum of approximately 1 × 10^8^ CFU/gland after 16 h (Figure 4). The administration of the extract that was done twice (2 × 300 μg/gland) at 0 h and 4 h post-infection did not significantly reduce the bacterial load when compared to the untreated control, although the bacterial counts in many of the treated glands showed lower CFU counts. On the other hand, the number of bacteria was significantly reduced (*P* < 0.001) to approximately 10^6^ CFU/gland by a treatment with pirlimycin (1 μg/gland at 4 h post-infection). Interestingly, there was an additional significant antibacterial effect when pirlimycin was used in combination with the RTL extract (*P* < 0.0001) when compared to the PBS control group. However, in those experimental conditions, there was no significant difference between treatment with pirlimycin alone or when in combination with the extract, although the bacterial counts in some of the glands treated with the combination reached the lowest CFU counts (Figure 4). Almost identical results were obtained when mice were challenged with strain Newbould (data not shown).

These results show that the RTL extract used at such a concentration and without any specific pharmacological formulation has some but limited activity in this mouse mastitis model. To that effect, we have determined the MIC of the RTL extract and of other antibiotics in the presence of 5% casein to mimic the milk environment. The results demonstrated that the addition of casein raised the MIC and MBC of the RTL extract since a fourfold loss of the activity was observed (data not shown). On the other hand, pirlimycin showed no reduction of the activity in presence of casein.

## Discussion

Considering that intramammary infections (IMIs) are responsible for most of the antibiotic use in dairy farms and that bacterial resistance to traditional antibiotics continues to progress and spread, the growing consumer concerns about food-related health risks have prompted the search for antibiotic alternatives for prevention and treatment of bovine mastitis. Since many medicinal plants have traditionally been used for remedy of some infectious diseases, plant extracts represent interesting sources of novel antibacterial substances.

In the present study, we are reporting the antibacterial effects of an ethanolic extract of *R. tomentosa* leaves (RTL), a Thai traditional herbal plant, and of rhodomyrtone, a compound purified from this plant, against staphylococcal strains associated with bovine mastitis. The RTL extract and purified rhodomyrtone provided a strong antibacterial activity against staphylococci strains with MICs of 8–16 and 0.25–0.5 µg/mL, respectively, including against *S. aureus* SCV and MRSA strains. The MICs obtained in the present study can be advantageously compared the other purified compounds from plant products or plant-derived antimicrobials. For instance, salvipisone, a diterpenoid compound isolated from the hairy roots of *Salvia sclarea*, has previously shown antibacterial activity against *S. aureus* and *S. epidermidis* with MIC values ranging from 9.37 to 18.75 µg/mL [26], whereas berberine, isolated from *Coptidis rhizome*, exhibited a bacteriostatic effect on *S. epidermidis* strains with MICs of 64 to 256 µg/mL [27]. Another example is thymoquinone, an active principle found in *Nigella sativa* seeds, which showed MICs against Gram-positive cocci (including *S. aureus* and *S. epidermidis*) ranging from 8 to 32 μg/mL [28].

Together with the growth inhibitory effect, other properties may be required from antibacterial substances in order to control pathogens like *S. aureus*, which possesses multiple strategies to colonize and persists in the bovine mammary gland. Here, we have also observed that the RTL extract and rhodomyrtone reduce internalization of *S. aureus* into bovine epithelial mammary cells, and that the RTL extract can improve the activity of some traditional antibiotics. While the mechanisms underlying such observations are yet to be identified, it is known that the RTL extract can affect the staphylococcal cells by increasing cell surface hydrophobicity in a concentration dependent manner [19]. Also, other pharmacological activities of the RTL extract and rhodomyrtone have been evaluated and documented in previous studies. For instances, the plant extract displayed a strong anti-biofilm forming effect against staphylococci [29], an anti-quorum sensing activity against *Streptococcus pyogenes* [30], as well as anti-oxidative [31] and anti-inflammatory properties [32]. Moreover, rhodomyrtone can enhance the killing activities of human monocytes and induce the expression of local host immunity during *S. aureus* infections [33]. All of these RTL extract or rhodomyrtone properties may thus act synergistically to combat *S. aureus* infections.

The ease of production of the RTL extract compared to the steps required for the purification of rhodomyrtone was a major motivation for the realization of this study. Both, the extract and pure compound, have a similar antibacterial profile in terms of MIC/MBC ratios and spectrum of activity, in accordance to the fact that the RTL extract contains a diluted amount of rhodomyrtone. However, some interesting differences were observed as no synergy with antibiotics was found with pure rhodomyrtone. Also, the toxicity of the pure compound toward mammalian cells suddenly increased at a certain concentration threshold but, for the extract, a more linear dose–response effect was observed. While rhodomyrtone represents at least one of the bioactive compounds, the RTL extract likely contains other bioactive compounds which may contribute to the various antibacterial, antioxidant and anti-inflammatory effects reported for the extract.

Experiments were carried out to determine the therapeutic effects of the RTL extract on *S. aureus* in a mouse mastitis model of infection. As sometimes seen with other natural products or extracts, the RTL extract was not as effective as expected in vivo and will likely need to be formulated to demonstrate its full antibacterial activity in a complex environment like the mammary gland. Many factors could have accounted for the low in vivo efficacy, but we found that the presence of milk casein might be sufficient to reduce the antibacterial effect of the RTL extract, and this possibly through casein binding to active RTL components.

In summary, the extract and the pure compound could reduce the ability of *S. aureus* SCVs to invade and survive intracellularly as shown by the reduced number of bacteria found inside bovine mammary epithelial cells after treatment. Moreover, a sub-MIC of the extract demonstrated an improved bactericidal activity when in combination with a sub-MIC of pirlimycin against *S. aureus* in vitro. While a proper pharmacological formulation would seem to be required to reveal the full activity of the RTL extract in vivo, our work show promising results that could qualify the extract as a possible natural alternative to traditional antibiotics or at the least as an agent that could help reduce antibiotic use in dairy farms. This view is in agreement with the conclusions of the 2^nd^ International Symposium on Alternatives to Antibiotics, which were recently reviewed by Lillehoj et al. [34] and which highlight the potential role and importance of phytochemicals in the global efforts aimed at reducing antibiotic use in animal productions.

## Data Availability

The datasets used and/or analyzed during the current study are available from the corresponding author on reasonable request.

## Abbreviations

ATB: antibiotic
BSA: bovine serum albumin
CFU: colony forming unit
CNS: coagulase negative staphylococci
DMEM: Dulbecco’s Modified Eagle’s Medium
DMSO: dimethyl sulfoxide
D-PBS: Dulbecco’s phosphate-buffered saline
FBS: fetal bovine serum
FIC: fractional inhibitory concentration
IMI: intramammary infection
LDH: lactate dehydrogenase
MBCs: minimum bactericidal concentrations
MHA: Mueller–Hinton agar
MHBCA: cation-adjusted Mueller–Hinton broth
MIC: minimal inhibitory concentration
MOI: multiplicity of infection
MRSA: methicillin-resistant *Staphylococcus aureus*
MSA: mannitol salt agar
OD: optical density
PBS: phosphate buffered saline
PIR: pirlimycin
RTL/RTLex: *Rhodomyrtus tomentosa* ethanolic leaf extract
SCV: small colony variant
TSA: tryptic soy agar
TSB: tryptic soy broth
ΣFIC: fractional inhibitory concentration index
